# Total colonic aganglionosis and cleft palate in a newborn with Janus-cysteine 618 mutation of *RET* proto-oncogene: a case report

**DOI:** 10.1186/s13052-020-00901-9

**Published:** 2020-09-18

**Authors:** Ingrid Anne Mandy Schierz, Marcello Cimador, Mario Giuffrè, Claudia Maria Aiello, Vincenzo Antona, Giovanni Corsello, Ettore Piro

**Affiliations:** 1 Neonatal Intensive Care Unit, Department of Health Promotion, Mother-Child Care, Internal Medicine and Medical Specialties “G. D’Alessandro”, University Hospital “P. Giaccone”, Via Alfonso Giordano n. 3, 90127 Palermo, Italy; 2 Pediatric Surgery Unit, Department of Health Promotion, Mother-Child Care, Internal Medicine and Medical Specialties “G. D’Alessandro”, University Hospital “P. Giaccone”, Via Alfonso Giordano n. 3, 90127 Palermo, Italy

**Keywords:** Case-report, *RE*arranged during *T*ransfection, Neurocristopathy, Hirschsprung disease, Congenital digestive system abnormalities

## Abstract

**Background:**

Hirschsprung disease, the most important congenital colonic dysmotility in children results from neural crest migration, differentiation, proliferation, or apoptosis defects where the rearranged during transfection (*RET*)-Protooncogene pathway has a central role. Although palatal and retinal anomalies in the context of chromosomopathies and some mono−/oligogenic syndromes are reported associated with Hirschsprung disease the role of inactivating RET mutations in these cases is not clarified.

**Case presentation:**

We report on a dysmorphic newborn with cleft palate and palatal synechia, who showed intestinal obstruction after 24 h of life. Transient ileostomy and surgical biopsies were performed to diagnose aganglionosis of the colon and last ileal loop. No chromosomal anomalies or copy number variations were found. We identified a paternal heterozygous germline mutation c.1852 T > C, which results in the substitution of cysteine by arginine in the RET-receptor tyrosine kinase (p.C618R mutation). There was no family history of Hirschsprung disease, but the father underwent surgery for medullary thyroid carcinoma and was affected by retinal dystrophy.

**Conclusions:**

The occurrence of Hirschsprung disease and carcinoma shows how a single mutation may be responsible for adverse effects: gain and loss of function of the same receptor. Furthermore, it would be interesting to study its dual role in face and retina embryology, and to extend targeted investigations of *RET* hotspots in these developmental abnormalities to facilitate counselling, follow-up, and tumor prevention. Complex surgical procedures and genetic testing as well as socio-economic impact are a challenge for familiar compliance.

## Background

Hirschsprung disease (HSCR, ♯142,623), a colonic dysmotility due to neural crest migration, differentiation, proliferation or apoptosis defects during intestinal development, occurs in approximately 1:5000 live births, more frequently in males (4:1), except for the long-segment-disease (1:1) [1, 2]. Especially the long-segment-disease (aganglionosis beyond the retrosigmoidal junction) is presenting during the first few days of life with features of intestinal obstruction and complications as perforation peritonitis or enterocolitis and has a poor outcome despite of timely surgical intervention [3].

As HSCR is a highly heritable neurocristopathy, genetic variation in the genomes of these patients must largely explain disease development. Inactivating *RE*arranged during *T*ransfection (*RET*, ♯164,761) mutations are implicated in approximately half the familial cases but also in 20% of sporadic cases, the majority of which were associated with long-segment-disease [2].

The gene *RET* encodes a transmembrane receptor tyrosine kinase, that is activated by a complex consisting of a soluble glial cell line-derived neurotrophic factor (GDNF) family ligand (GFL) and a glycosyl phosphatidylinositol-anchored co-receptor, GDNF family receptors alpha (GFRα). Four different GFLs, namely GDNF, neurturin, artemin and persephin, can bind to and specifically activate RET through their cognate coreceptors GFRα1–4, respectively [1, 4]. As a signal transducer of these four different ligand/co-receptor complexes, RET has many functions in different tissues and mediates signals through a range of pathways including RAS/ERK, p38MAPK, NF-κB, PI3/AKT, and JNK.

Chromosomopathies (Down syndrome, Cat-eye-syndrome) and some mono- or oligogenic syndromes (Bardet-Biedl syndrome, Cartilage-hair hypoplasia, Goldberg-Shprintzen syndrome, Ondine-Hirschsprung syndrome, Mowat-Wilson syndrome, Smith-Lemli-Opitz syndrome, Waardenburg-Shah syndrome) occasionally include palatal and retinal anomalies and are frequently associated with HSCR due to phenotype modifier effects of *RET* haplotypes [1, 4–6] (Table 1).

**Table 1:** Chromosomal loci with risks of Hirschsprung disease, modified [6, 7]

Cytogenetic location/gene	Relative risk	Prevalence in HSCR	Mode of inheritance	Phenotype
1p36.12/*ECE1*		rare	AD	HSCR, cardiac defects, autonomic dysfunctions
1q43-q44/*SDCCAG8*			AR	Bardet-Biedl syndrome-16 (like Bardet-Biedl syndrome-1)/Senior-Loken syndrome-7
2q22.3/*ZFHX1B (ZEB2)*	3000-fold	6%	AD	HSCR/Mowat-Wilson syndrome (MR, craniofacial dysmorphisms)
2q31.1/*BBS5*			AR	Bardet-Biedl syndrome-5 (like Bardet-Biedl syndrome-1)
3p21	4-fold			HSCR6
3p21.32/*CELSR3 (EGFL1)*				Encephalopathy
3p21.31/*LZTFL1*			AR	Bardet-Biedl syndrome-17 (like Bardet-Biedl syndrome-1)
3q11.2/*ARL6*			AR	Bardet-Biedl syndrome-3 (like Bardet-Biedl syndrome-1)
4p13/*PHOX2B*	1000-fold	0.5%	AD	Ondine syndrome (hypoventilation, autonomic dysfunctions)
4q27/*BBS7*			AR	Bardet-Biedl syndrome-7 (like Bardet-Biedl syndrome-1)
4q27/*BBS12*			AR	Bardet-Biedl syndrome-12 (like Bardet-Biedl syndrome-1)
4q31.3-q32.3				HSCR9
5p13.2/*GDNF*		rare	AD	HSCR3/Ondine syndrome (hypoventilation, autonomic dysfunctions)
7p14.3/*PTHB1*			AR	Bardet-Biedl syndrome-9 (like Bardet-Biedl syndrome-1)
7q21.11/*SEMA3A/SEMA3D*	1.5-fold		AD	Hypogonadism, anosmia, altered axon branching
8p12/*NRG1*	1.2-fold		AD	Schizoaffective disorders
8q22.1/*C8orf37*			AR	Bardet-Biedl syndrome-21 (like Bardet-Biedl syndrome-1)
8q24.3/*DENND3*				neurological involvement
9p13.3/*RMRP*	25-fold	0.5%	AR	Cartilage-hair hypoplasia (short limb dwarfism, hypotrichosis, immunodeficiency)
9p21.2/*IFT74*			AR	Bardet-Biedl syndrome-20 (like Bardet-Biedl syndrome-1)
9p24.1				
9q31/*IKBKAP*			AD; AR	HSCR5; Riley-Day syndrome (sensory and autonomic dysfunction)
9q33.1/*TRIM32*			AR	Bardet-Biedl syndrome-11 (like Bardet-Biedl syndrome-1)
10q11.21/*RET*	3000-fold	48% of familial 20% of sporadic	AD	HSCR1 classical; HSCR/medullary thyroid carcinoma/multiple endocrine neoplasia syndrome-2A/−2B/ Ondine syndrome (hypoventilation, autonomic dysfunctions)
10q22.1/*C10orf27 (TBATA)*				Thymus, brain and testes associated (TBATA) alterations
10q22.1/*KIAA1279 (KIF1BP)*			AR	Goldberg-Shprintzen megacolon syndrome (MR, craniofacial dysmorphisms, nervous system anomalies)
10q22.2/*VCL*			AD	Myopathy
10q23.1/*NRG3*				Schizoaffective disorders
10q25.2/*BBIP1*			AR	Bardet-Biedl syndrome-18 (like Bardet-Biedl syndrome-1)
10q25.3/*GFRA1*		rare		HSCR
11p15.4/*NUP98*				myelodysplastic syndrome
11q13.2/*BBS1*			AR, DR	Bardet-Biedl syndrome-1 (MR, obesity, retinal degeneration, genitourinary anomalies, HSCR, polydactyly and other laterality defects)
11q13.4/*DHCR7*	50-fold	1%	AR	Smith-Lemli-Opitz syndrome (MR, craniofacial dysmorphisms, 2–3 toe syndactyly, multiple malformations, hypogonadism)
12q21.2/*BBS10*			AR	Bardet-Biedl syndrome-10 (like Bardet-Biedl syndrome-1)
12q21.32/*CEP290*			AR	Bardet-Biedl syndrome-14 (like Bardet-Biedl syndrome-1)/Joubert-syndrome/Meckel syndrome/Leber congenital amaurosis-10
12q23.2/*ASCL1*			AD	Ondine syndrome (hypoventilation, autonomic dysfunctions)
13q22.3/*EDNRB*	1000-3700-fold	5%	AD, AR	HSCR2/ABCD syndrome/ Waardenburg-syndrome-4A (deafness, pigmentation defects)
14q13.3/*NKX2–1*			AD	Nonmedullary thyroid carcinoma, brain-lung-thyroid syndrome, chorea
14q31.3/*TTC8*			AR	Bardet-Biedl syndrome-8 (like Bardet-Biedl syndrome-1)
15q24.1/*BBS4*			AR	Bardet-Biedl syndrome-4 (like Bardet-Biedl syndrome-1)
16q13/*BBS2*			AR	Bardet-Biedl syndrome-2 (like Bardet-Biedl syndrome-1)
16q23				HSCR8
17q11.2/*NF1*			AD	Neurofibromatosis 1 (Cafè-au-lait macules, neurofibromas, Lisch nodules)
17q21/*HOXB5 (HOX2A)*				
17q22/*MKS1*				Bardet-Biedl syndrome-13 (like Bardet-Biedl syndrome-1)/Joubert-syndrome/Meckel syndrome
19p13.3/*NRTN*		rare		HSCR
19p13.3/*NCLN*				neurological involvement
19q12	5-fold			HSCR7
20p11.22-p11.23				
20p12.2/*MKKS*			AR	McKusick-Kaufman/Bardet-Biedl syndrome-6 (like Bardet-Biedl syndrome-1)
20q13.32/*EDN3*		rare	AD, AR	HSCR4/Waardenburg-syndrome-4B (deafness, pigmentation defects); Ondine syndrome (hypoventilation, autonomic dysfunctions)
Trisomy 21 (21q22)	50–100-fold	8%		Down syndrome (MR, craniofacial dysmorphisms, gastrointestinal and cardiac malformations)
Partial tetrasomy 22q11			AD	Cat eye syndrome (coloboma, craniofacial dysmorphisms, anorectal and cardiac malformations)
22q12.3/*IFT27*			AR	Bardet-Biedl syndrome-19 (like Bardet-Biedl syndrome-1)
22q13.1/*SOX10*	> 4000-fold	4%	AD	HSCR/Waardenburg syndrome-4C or -2E (deafness, anosmia, pigmentation defects, hypogonadism, neurological involvement)
Xq28/*L1CAM*	40-fold	1%	XLR	L1 syndrome (MR, hydrocephalus due to congenital stenosis of aqueduct of Sylvius, adducted thumbs)
Male sex	4-fold	80% short-segment 65% long-segment		- SRY competes for SOX10 binding site - Males have less ECE1 and EDN3 expression in bowel

Other genes (*ABCC9, ARID1B, ARVCF, BACE2, COMT, CREBBP, DLL3, DNMT3B, DSCAM, ELP1,FZD3, GABRG2, GAL, GAP43, GHRL, IL11, INMT, MAPK10, MBTPS2, MED12, MIR146A, MIR369, NRSN1, NTRK3, PIGV, PSPN, PTCH1, RELN, RORA, SAMD9, SLC6A20, SOX2, TCF4, TUBA1A, VAMP5, WNT3A*) without direct evidence for role in HSCR are actually classified as red/research gene only (https://panelapp.genomicsengland.co.uk/)

Abbreviations: *AD* Autosomal dominant, *AR* Autosomal recessive, *ARL6* ADP-ribosylation factor-like protein 6 gene, *ASCL1* Achaete-scute homolog 1, *BBIP1* BBSome interacting protein 1 gene, *BBS* Bardet Biedl syndrome gene, *CELSR3* Cadherin EGF LAG seve-pass G-type receptor 3 gene, *CEP290* Centrosomal protein gene, *C8orf37* Chromosome 8 open reading frame 37, *C10orf 27* Chromosome 10 open reading frame 27, *DENND3* DENN domain-containing protein 3 gene, *DHCR7* 7-dehydrocholesterol reductase gene, *DR* Digenic recessive, *EDNRB* Endothelin receptor type b gene, *EDN3* Endothelin 3 gene, *GDNF* Glial cell line-derived neurotrophic factor gene, *GFRA1* GDNF family receptor alpha-1, *HSCR* Hirschsprung disease, *IFT* Intraflagellar transport, *IKBKAP* Inhibitor of kappa light polypeptide gene enhancer gene, *KIAA1279* Kinesin binding protein*, LZTFL1* Leucine zipper transcription factor like 1, *L1CAM* L1 cell adhesion molecule gene, *MKKS* McKusick-Kaufman syndrome gene, *MKS1* Meckel syndrome-1 gene, *MR* Mental retardation, *NCLN* Nicalin, *NF1* Neurofibromin 1 gene, *NKX2–1* NK1 Homebox 1, *NRG1* Neuregulin 1, *NRTN* Neurturin, *NUP98* Nucleoporin 98, *PHOX2B* Paired-like homeobox 2B gene, *PTHB1* Parathyroid hormon-responsive B1 gene, *RET* Rearranged during transfection protooncogene, *RMRP* RNAse mitochondrial RNA processing gene, *SDCCAG8* Serologically defined colon cancer antigen 8 gene, *SEMA* Semaphorin, *SOX10* Sry-box 10 gene, *TTC8* Tetratricopeptide repeat domain 8, *TRIM32* Tripartite motif containing 32, *VCL* Vinculin, *XLR* X-linked recessive, *ZFHX1B* Zinc finger homeobox protein 1b gene

Orofacial clefts (cleft lip and/or cleft palate) are the result of tissues of the face not joining properly during development, occur in up to 1:690 live births, and are twice as frequent in males [8, 9]. Cleft palate alone occurs when midline fusion of the palatal shelves fails, has a frequency of 1:1500, and is prevalent in female neonates [9]. Advanced maternal age, some maternal medications, cigarette smoking and folate deficiency have been associated with the risk of isolated orofacial clefts in offsprings [9]. Although of complex heterogenetic origin, syndromic cleft palate might be associated with variants in a single gene or in a cluster of contiguous genes (copy number variants), such as van der Woude syndrome, 22q11-deletions syndrome, or chromosomopathies [9, 10]. Furthermore, in non-syndromic orofacial clefts more than 50 genes as well as additive gene-gene and gene-environment interactions, with modifier phenotypic effects, are postulated to play an etiologic role. We present herein a family in which the father manifested medullary thyroid carcinoma (MTC) and the infant not only HSCR but also cleft palate as a developmental abnormality by the loss-of-function nature of Janus-Cysteine 618 mutation of *RET* proto-oncogene.

## Case presentation

The boy was born at full term from non-consanguineous Caucasian parents and was spontaneously delivered without perinatal problems and with weight (3440 g) appropriate for his gestational age. His mother aged 36 years and his father 39 age, and both were originating from a small Greek-spoken southern Sicilian (Italy) village. He was referred to our Institution for mild hypotonia and bilateral cleft of the hard and soft palate (Veau-II cleft palate). Other clinical findings included prominent forehead, a filiform synechia between lingual frenulum and anterior hard palate and syndactyly of second and third toes of left foot. The single synechia was long enough for him to be able to open the mouth. The patient did not have other anomalies of the midline nor congenital lip pits or limb or skeletal malformations.

**Fig. 1:**
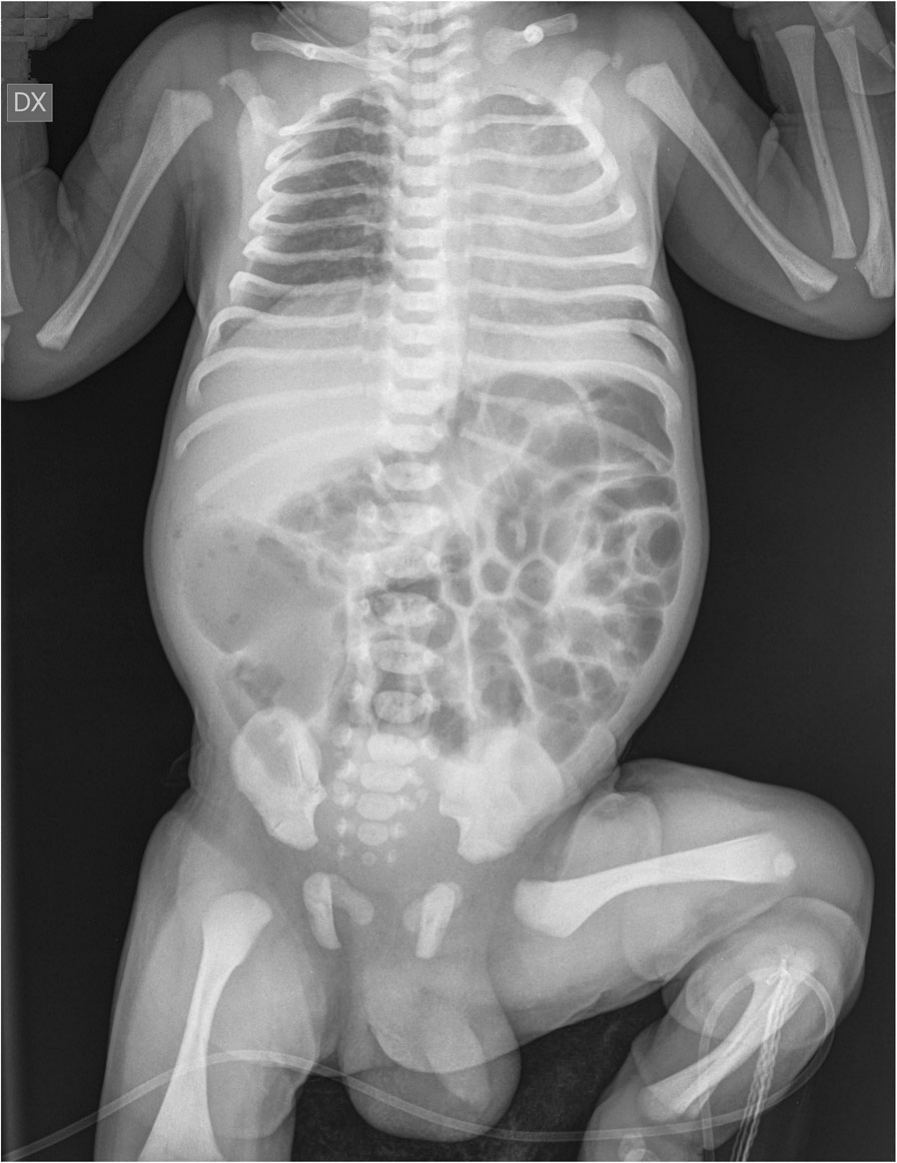
Plain abdominal radiogram shows distended small bowel loops, distal obstruction, and absent rectal gasification, consisting with total colonic aganglionosis. There are no peritoneal free fluid or air and no associated skeletal anomalies or maturation defects, except for the still absent first coccygeal ossification center

Enteral nutrition was replaced with parenteral nutrition after 24 h because of clinical evidence of bowel obstruction. The meconium did not pass, he had biliary emesis and abdominal distension. Radiographical investigations showed distended small bowel loops with a distal obstruction (Fig. 1). Laboratory and ultrasound studies were unremarkable. On third day of life, he underwent an explorative laparotomy and a double terminal ileostomy. Immunohistochemical examination showed in all colonic and distal ileal biopsies the absence of ganglion cells in the intestinal nerve plexus consisting with HSCR.

A laparoscopic assisted ileoanal endorectal pull-through and a palatoplasty and synechia release have been planned at 8 and 15 months of age, respectively. The synechia could be useful to provide additional tissue for the following surgical closure of soft palate.

The family history was negative for HSCR, but the father underwent a thyroidectomy at the age of 38 years for an accidentally diagnosed MTC and a germline *RET* proto-oncogene mutation (p.C618R).

The father presents also hypernasal speech and a not better investigated inherited retinal dystrophy.

Based on clinical presentation and history the newborn’s family followed genetic counselling and periodically check-up for onset of neural crest tumors. The infant’s cardiac, renal, neurodevelopmental, and ophthalmological examinations were unremarkable at 7 months of age.

Laboratory investigations in the newborn were performed. High-resolution GTG-banding karyotype excluded aneuploidies. Deoxyribonucleic acid (DNA) extraction from lymphocytes (QIAamp DNA blood Midi Kit, Qiagen) was followed by an array comparative genomic hybridization (a-CGH) using the whole genome 8x60K. Scanned images of the arrays were processed and analyzed using Feature Extraction software and Genomic Workbench software (Agilent Technologies) with the statistical algorithm Aberration detection method-2 (ADM-2) and a sensitivity threshold of 6.0 as recently benchmarked [7]. This genetic analysis did not reveal cryptic chromosomal anomalies associated with HSCR or cleft palate.

Polymerase chain reaction amplification of the *RET* proto-oncogene (♯164,761) on 10q11.2 followed by bi-directional direct sequencing (GenBank NM_020975.4) identified the paternal heterozygous germline mutation c.1852 T > C on Exon 10 also in the newborn, which results in the known substitution of cysteine by arginine in the RET receptor (p.C618R). Informed parents refuted to proceed with further studies involving other genes.

## Discussion and conclusion

We observed an association of total colonic aganglionosis and cleft palate with palatal synechia in a newborn with paternal Janus-Cysteine 618 mutation of *RET* proto-oncogene and familial history of MTC.

Actually, HSCR cannot be diagnosed in utero, but it could be suspected if positive family history and early complications as meconium peritonitis occur. It is commonly diagnosed shortly after birth by image-based and non–image-based clinical techniques and specific laboratory tests to detect ganglion cells and nerve fibers on suction biopsies. These biopsies stained with hematoxylin and eosin and/or acetylcholinesterase are showing absent submucosal ganglion cells and an increase in nerve fibers in the submucosa and an increase in nervous filaments in the lamina propria. Combined immunohistochemical markers, as calretinin, S-100 protein, peripherin, neuron-specific enolase, cathepsin D, BCL-2 (B-cell lymphoma 2) and RET have been described as adjunctive diagnostic tests in HSCR. Genetic markers in familiar forms could be helpful also for prognosis.

Ligand-independent activating mutations of *RET* cause neural crest proliferation defects in single (MTC, ♯155,240) or multiple sites (Multiple endocrine neoplasia type 2A, ♯171,400; Multiple endocrine neoplasia type 2B, ♯162,300), while 2–5% of patients with HSCR also develop MTC [2, 11]. In case of this rare co-segregation, dual Janus mutations in exon 10 (codons 609, 611, 618, and 620) are implicated affecting the Cysteine-rich region of RET receptor by homodimerization. The closer mutations are located to the transmembrane domain of RET, the higher their tumorgenicity in thyroid parafollicular cells and adrenal chromaffin cells, and the lower the density of RET on the cell surface leading to apoptosis in precursor neurons in the developing enteric nervous system [12, 13].

While *RET* is crucial during embryogenesis, since it is expressed in all neural crest-derived cells and renal epithelium, its inactivating mutations could explain cleft palate (propositus) and retinal dystrophy (father) [13]. To our knowledge, this association with *RET* mutations has never been reported.

Many genetic factors contributing to cleft palate formation have been identified for some syndromic cases. However, many clefts run in families even though in some cases there does not seem to be any identifiable syndrome present [14].

A large number of genes are involved in nonsyndromic forms of orofacial clefts, including above all growth factors (*CLPTM1*, *FGFR1, TGFA, TGFB3*), genes related to nutritional metabolism (*GAD1*, *LRP6, MTHFR*) and transcription factors (*GRHL3, IRF6, MSX1, TBX1, TBX22, TP63*) [10, 14, 15]. Unfortunately, we could not further investigate these genes because the parents refused any further genetic examination. However, clinically and genetically we could exclude the most frequent monogenetic or copy-number variations associated with palatal and retinal anomalies and HSCR [6, 7, 15]. Congenital intraoral synechiae associated with an oral cleft are exceedingly rare, described as clinical conditions as cleft palate lateral synechiae (CPLS) syndrome or cleft palate and congenital alveolar synechia (AS) syndrome [14, 16]. However, and association with HSCR without lower lip pits has not reported.

Up to date the etiology is unclear and interposition of the tongue between the palatal shelves explain the cleft palate, while close contact between the floor of the mouth and the palate could predispose to the formation of the subglossopalatal membrane a precursor of intraoral synechiae. We have been able to exclude the most frequent syndromic forms. Patient did not resemble the autosomal-dominant van der Woude syndrome because the pathognomonic characteristics of lower lip pits or pyramidal-shaped skin above the big toe were missing in the neonate and his parents [14]. He did also not have any other reported phenotypic features of orofaciodigital syndromes, or extensive webbing behind the knee characteristic for autosomal-dominant popliteal pterygium syndrome, or any musculoskeletal or thoracic anomalies described in autosomal-recessive Fryns syndrome [9, 13].

Since HSCR most often presents shortly after birth, features of correlated syndromes may not be reported at the time of diagnosis. We could exclude consanguinity associated with hypothetical rare recessive disorders; however, oblivious additional heterozygosity conditions could be presumed since parents originate from the same small Greek-spoken Sicilian territory (Siceliots). Furthermore, just the p.C618R mutation is considered a founder mutation in this Northern Southwest Asian J2 haplogroup which is spreading also among Greek Cypriots [17, 18]. Gene-environment and gene-gene interactions, as epigenetic modifiers, are the hallmark of inconstant penetrance, parent-of-origin effects, and more insidious disease in males than females in HSCR and MTC but could also open to future therapeutic perspectives [4–6, 18, 19].

In the interests of cost-effectiveness, targeted investigations of *RET* hotspots in HSCR patients should facilitate counselling, follow-up, and tumor prevention.

Complex surgical procedures and genetic testing as well as socio-economic impact are a challenge for familiar compliance.

## Data Availability

The datasets used and/or analyzed during the current study are available from the corresponding author on reasonable request.

## Abbreviations

a-CGH: Array-comparative genomic hybridization
ADM-2: Aberration detection method-2
DNA: Deoxyribonucleic acid
GDNF: Glial cell line-derived neurotrophic factor
GFL: Glial cell line-derived neurotrophic factor family ligand
GFRα: Glial cell line-derived neurotrophic factor family receptors alpha
HSCR: Hirschsprung disease
MTC: Medullary thyroid carcinoma
RET: Rearranged during transfection

## References

[1] Amiel J, Sproat-Emison E, Garcia-Barcelo M, Lantieri F, Burzynski G, Borrego S (2008). Hirschsprung disease, associated syndromes and genetics: a review. J Med Genet.

[2] Vaclavikova E, Kavalcova L, Skaba R, Dvorakova S, Macokova P, Rouskova B (2012). Hirschsprung’s disease and medullary thyroid carcinoma: 15-year experience with molecular genetic screening of the RET proto-oncogene. Pediatr Surg Int.

[3] Sarin YK, Raj P, Thakkar N (2014). Perils of Total colonic Aganglionosis presenting in neonatal age. J Neonatal Surg.

[4] de Pontual L, Pelet A, Clement-Ziza M, Trochet D, Antonararkis SE, Attie-Bitach T (2007). Epistatic interactions with a common hypomorphic RET allele in syndromic Hirschsprung disease. Hum Mutat.

[5] Fitze G, Cramer J, Ziegler A, Schierz M, Schreiber M, Kuhlisch E (2002). Association between c135G/a genotype and RET proto-oncogene germline mutations and phenotype of Hirschsprung’s disease. Lancet..

[6] Heuckeroth RO (2018). Hirschsprung disease - integrating basic science and clinical medicine to improve outcomes. Nat Rev Gastroenterol Hepatol.

[7] Lantieri F, Malacarne M, Gimelli S, Santamaria G, Coviello D, Ceccherini I (2017). Custom Array comparative genomic hybridization: the importance of DNA quality, an expert eye, and variant validation. Int J Mol Sci.

[8] Dixon MJ, Marazita ML, Beaty TH, Murray JC (2011). Cleft lip and palate: understanding genetic and environmental influences. Nat Rev Genet.

[9] Impellizzeri A, Giannantoni I, Polimeni A, Barbato E, Galluccio G (2019). Epidemiological characteristic of Orofacial clefts and its associated congenital anomalies: retrospective study. BMC Oral Health.

[10] Maili L, Letra A, Silva R, Buchanan EP, Mulliken JB, Greives MR (2020). PBX-WNT-P63-IRF6 pathway in nonsyndromic cleft lip and palate. Birth Defects Res.

[11] Wells SA, Asa SL, Dralle H, Elisei R, Evans DB, Gagel RF (2015). American Thyroid Association guidelines task force on medullary thyroid carcinoma. Revised American Thyroid Association guidelines for the management of medullary thyroid carcinoma. Thyroid..

[12] Arighi E, Popsueva A, Degl’Innocenti D, Borrello MG, Carniti C, Perälä NM (2004). Biological effects of the dual phenotypic Janus mutation of ret cosegregating with both multiple endocrine neoplasia type 2 and Hirschsprung’s disease. Mol Endocrinol.

[13] Moore SW (2006). The contribution of associated congenital anomalies in understanding Hirschsprung’s disease. Pediatr Surg Int.

[14] Basha M, Demeer B, Revencu N, Helaers R, Theys S, Bou Saba S (2018). Whole exome sequencing identifies mutations in 10% of patients with familial non-syndromic cleft lip and/or palate in genes mutated in well-known syndromes. J Med Genet.

[15] da Silva HPV, Oliveira GHM, Ururahy MAG, Bezerra JF, de Souza KSC, Bortolin RH (2018). Application of high-resolution array platform for genome-wide copy number variation analysis in patients with nonsyndromic cleft lip and palate. J Clin Lab Anal.

[16] Imai Y, Tachi M (2020). Congenital lateral palatal synechia associated with cleft palate: a case report with long-term follow-up and review of the literature. Cleft Palate Craniofac J.

[17] Neocleous V, Skordis N, Portides G, Efstathiou E, Costi C, Ioannou N (2011). RET proto-oncogene mutations are restricted to codon 618 in Cypriot families with multiple endocrine neoplasia 2. J Endocrinol Investig.

[18] Hibi Y, Okye T, Ogawa K, Shimizu Y, Shibata M, Kagawa C (2014). A MEN2A family with two asymptomatic carriers affected by unilateral renal agenesis. Endocr J.

[19] Sergi CM, Caluseriu O, McColl H, Eisenstat DD (2017). Hirschsprung’s disease: clinical dysmorphology, genes, micro-RNAs, and future perspectives. Pediatr Res.

